# Effectiveness and safety of tenofovir amibufenamide and tenofovir alafenamide in treating elderly patients diagnosed with decompensated hepatitis B cirrhosis: a retrospective cohort study

**DOI:** 10.3389/fphar.2025.1545108

**Published:** 2025-04-03

**Authors:** Xinye Qiu, Yue Yin, Shibin Zhang, Wei Liu

**Affiliations:** ^1^ Department of Pharmacy, Beijing Youan Hospital, Capital Medical University, Beijing, China; ^2^ Key Laboratory of Carcinogenesis and Translational Research (Ministry of Education/Beijing), Department of Pharmacy, Peking University Cancer Hospital & Institute, Beijing, China; ^3^ Hepatology and digestion Center, Beijing Youan Hospital, Capital Medical University, Beijing, China

**Keywords:** tenofovir amibufenamide (TMF), tenofovir alafenamide (TAF), elderly patients, decompensated hepatitis B cirrhosis, virologic response (VR)

## Abstract

**Background/aim:**

Tenofovir amibufenamide (TMF) has demonstrated significant antiviral activity and safety in individuals with chronic hepatitis B (CHB) in randomized clinical trials. The purpose of this study was to investigate the effectiveness and safety disparities between TMF and Tenofovir alafenamide (TAF) in treating elderly patients with decompensated hepatitis B cirrhosis in real-world settings.

**Methods:**

A retrospective cohort analysis of elderly patients with decompensated hepatitis B cirrhosis who were treated with TMF or TAF in our hospital’s outpatient department between January 2022 and December 2023 was the focus of this study. Following a 24-week treatment period, this study evaluated the disparities between the TMF and TAF groups in terms of primary efficacy endpoints (virologic response rate, VR rate), secondary efficacy endpoints (normalization rate of ALT, HBsAg and HBeAg clearance rate, HBsAg and HBeAg seroconversion rates), as well as safety endpoints related to renal function and blood lipids.

**Results:**

The study included 171 patients (93 in the experimental group and 78 in the control group). Following a 24-week treatment period, HBV DNA, HBsAg, ALT, AST, and TBIL had significantly decreased compared to the baseline level, and the differences were statistically difference. Cr, eGFR, triglyceride, and TG had no significant changes compared with the baseline level, and the differences were no statistical difference. The virologic response rate in the experimental group was 70.97% (33/93), and that in the control group was 73.08% (57/78), with no statistical difference observed between the two groups (P = 0.760). ALT normalization rate was 83.33% in the experimental group and that was 100% in the control group, and there was not a statistically significant distinction between the two groups (P = 0.229). Compared with baseline data, Cr and eGFR of the experimental group increased (2.97 ± 14.66 μmol/L, P = 0.867; 0.29 ± 6.76 mL/min/1.72 m^2^, P = 0.680), TC and TG decreased (−0.5 ± 1.30 mmol/L, P = 0.589; −0.006 ± 0.23 mmol/L, P = 0.986), however, no statistical difference was observed. Compared with the control group, the change of safety dates was also no statistical difference.

**Conclusion:**

TMF treatment in elderly patients with decompensated hepatitis B cirrhosis had a good antiviral effect, no adverse drug reaction on renal function and blood lipids, and high safety. TMF is not inferior to TAF in antiviral efficacy and safety.

## 1 Introduction

The Hepatitis B virus (HBV) infection constitutes a global epidemic, with a prevalence of hepatitis B surface antigen (HBsAg) reaching 3.8% in the general population in 2019, equating to around 296 million chronic infections (Global progress, 2021). The infection rate of HBV in China is significantly higher than globally. According to the Polaris International Epidemiology Cooperation Organization, in China, the prevalence rate of HBsAg in the general population was 6.1% in 2016, and there were about 86 million CHB cases, accounting for about 30% in the world (Polaris Observatory Collaborators, 2023). As per the recommendation of Guidelines for the Prevention and Treatment of Chronic Hepatitis B (2022 Edition) of the Chinese Medical Association (You et al., 2023), first-line antiviral therapy drugs include Entecavir (ETV), Tenofovir disoproxil (TDF), Tenofovir alafenamide (TAF), and Tenofovir amibufenamide (TMF), which have strong antiviral effects and a high HBV resistance barrier (Yim et al., 2020).

TMF is a Class Ⅰ novel drug independently developed in China (Liu et al., 2024). The drug is methylated on the basis of TAF and forms a monophosphamide monoester TFV prodrug through phosphoramide esterification prodrug technology, which improves TFV liver targeting, reduces peripheral TFV concentration, improves drug bioavailability, and reduces bone and kidney-related adverse drug reactions (Trépo et al., 2014). Phase III clinical trials have demonstrated that the antiviral efficacy of TMF is non-inferior to TDF, with superior safety profiles for bone and renal (Liu et al., 2021). Real-world studies suggest that TMF has fewer kidney-related adverse reactions, and its antiviral efficacy is non-inferior to TAF, and it is superior to TAF in treatment-naive patients (Li et al., 2023). However, there remains a deficiency of efficacy and safety evidence from clinical trials that persists in the real-world studies, and drug instructions specifically pertaining to patients with decompensated hepatitis B cirrhosis aged ≥65 years. Thus, this research aims to investigate the effectiveness and safety of 24-week TMF treatment in the real-world with patients with decompensated hepatitis B cirrhosis aged ≥65 years while comparing it against counterparts treated with TAF.

## 2 Materials and methods

### 2.1 Study design and patients

This study was a retrospective cohort analysis of patients treated with TMF or TAF in the outpatient department of our hospital between January 2022 and December 2023. Inclusion criteria: (1) Patients diagnosed with decompensated hepatitis B cirrhosis who meet the diagnostic criteria of the Chinese Medical Association’s Guidelines for the Prevention and Treatment of Chronic Hepatitis B (2022 edition) (You et al., 2023) and the Chinese Medical Association’s Guidelines for the Diagnosis and Treatment of Liver Cirrhosis (2019 edition) (Chinese Society of Hepatology and Chinese Medical Asociation, 2019); (2) Age ≥65 years old; (3) HBsAg positive; (4) treatment-naive (TN) patients who meet the antiviral indication of HBV or treatment-experienced (TE) patients who have received antiviral therapy with nucleoside (acid) analogues but have poor efficacy, as well as TE patients who have renal, bone and other related adverse drug reactions or are at risk of related adverse drug reactions; (5) TN patients need to be HBV DNA positive; (6) Treatment duration should be a minimum of 24 weeks. Exclusion criteria: (1) Patients possessing inadequate clinical data; (2) Pregnant or lactating women; (3) Hepatitis A virus (HAV) patients, hepatitis C virus (HCV) patients, hepatitis D virus (HDV) patients, hepatitis E virus (HEV) patients, acquired immune deficiency syndrome (AIDS) patients, drug-induced liver injury patients, autoimmune hepatitis (AIH) patients, primary biliary cholangitis (PBC) patients, primary sclerosing cholangitis (PSC) patients, alcoholic liver disease patients and liver transplantation patients; (4) Patients who do not take the drug according to the TMF instructions; (5) combination with other anti-HBV drugs, such as other nucleoside (acid) analogues or interferons; (6) interferon treated patients; (7) Patients undergoing immunosuppressive therapy. We categorized the study into an experimental group and a control group. Patients in the experimental group took 25 mg TMF orally once daily (Hansoh Pharmaceuticals Co., Ltd., Jiangsu, China). Control group patients took 25 mg TAF orally once daily (Patheon Inc., Ontario, Canada). The serum HBV detection kit uses “Abbott RealTime HBV Assay”.

This study rigorously followed the ethical guidelines of the 1975 Declaration of Helsinki and was approved by the Ethics Committee of Beijing Youan Hospital, Capital Medical University (serial number, LL-2024-137-K). Additionally, this study was registered in the Chinese Clinical Trial Registry (ChiCTR2400093201).

### 2.2 Endpoints

The main effectiveness endpoint was the virologic response (VR), defined as a serum HBV DNA level of less than 10 IU/mL at week 24. Secondary effectiveness endpoints were established as the ratio of normal ALT (male ALT ≤50 U/L, female ALT ≤40 U/L), HBeAg and HBsAg clearance, and HBeAg seroconversion from the baseline until the 24th week. The safety endpoints were alterations in renal function and blood lipids.

### 2.3 Statistical analysis

Continuous variables exhibiting normal distribution were presented as mean ± standard deviation (SD) ( $\bar{x}$ ±*s*) and analyzed using the independent samples t-test. The data from the non-normal distribution were presented as median and quartiles [M (Q1, Q3)] and analyzed using the Mann-Whitney U test. Categorical variables were represented as numbers (percentages) and analyzed using a chi-squared or Fisher’s exact test. All statistical tests indicated that p < 0.05 was significant. All statistical analyses were conducted with IBM SPSS software version 26.0.

## 3 Results

### 3.1 Baseline characteristics

**FIGURE 1:**
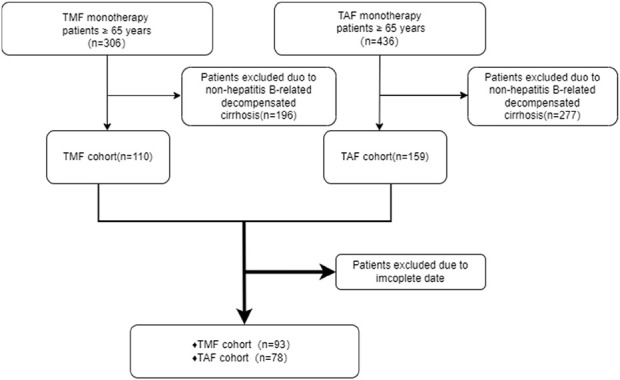
Flowchart of the study population.

A total of 171 patients were enrolled, which includes 93 in the experimental group and 78 in the control group (Figure 1). Table 1 presents the baseline characteristics of the enrolled patients. The positive rate of HBeAg in the experimental group was 35.48%, and the ratio of ALT in the normal range was 80.64%. In the control group, the positive rate of HBeAg was 46.15%, and the ratio of ALT in the normal range was 76.92%. In the data collected at the beginning of the study, there were no discernible differences between the two groups (P > 0.05) except for serum creatinine (Cr), estimated glomerular filtration rate (eGFR), total cholesterol (TG), and triglyceride (TC). The basal eGFR abnormality rate was 45.16% in the experimental group, and the control group exhibited a rate of 7.70%. The levels of TC and TG in the experimental group were substantially lower than those in the control group.

**TABLE 1:** Baseline data of the patients.

Project	Experimental group	Control group	Statistic	p-value
Gender,n			χ^2^ = 0.005	0.945
Male patients	60	51		
Male patients	33	27		
Age (years)	71.26 ± 4.31	70.23 ± 3.53	*t* = 0.971	0.336
HBeAg,n			χ^2^ = 2.007	0.157
Positive	33	36		
Negative	60	42		
History of antiviral treatment,n			χ^2^ = 3.290	0.707
treatment-naive	57	58		
treatment-experienced	36	20		
HBV DNA (IU/mL)	1110 (300,325000)	852 (40.5,111000)	*Z* = 0.721	0.471
HBsAg(IU/mL)	877 (287,1794)	470 (176,1853)	*Z* = 0.874	0.382
ALT (U/L)	32 (24,46)	23 (17,77)	*Z* = 1.211	0.226
AST (U/L)	41 (29,66)	39 (27,65)	*Z* = 0.120	0.904
TBIL (μmol/L)	24.6 (15.1,37.5)	21.9 (15.5,28.0)	*Z* = 0.184	0.854
Cr (μmol/L)	71.10 ± 24.85	60.27 ± 13.16	*t* = 2.007	0.049
eGFR (mL/min/1.72m^2^)	92.80 (83.20,96.10)	96.20 (92.60,99.25)	*Z* = 2.356	0.018
TC (mmol/L)	3.99 (2.93,4.59)	5.08 (4.09,5.52)	*Z* = 3.015	0.003
TG (mmol/L)	0.95 (0.75,1.23)	1.33 (1.12,1.59)	*Z* = 2.815	0.005

^a^ TBIL, total bilirubin; AST, aspartate aminotransferase; ALT, alanine aminotransferase; Cr, creatinine; eGFR, estimated glomerular filtration rate; TC, total cholesterol; TG, triglyceride.

### 3.2 TMF effectiveness

#### 3.2.1 Laboratory examination

As shown in Table 2, after 24 weeks of follow-up after TMF treatment, HBV DNA, HBsAg level, ALT, AST, and TBIL of patients were significantly reduced compared with the treatment baseline, with statistical significance (p < 0.05). Renal function indexes (Cr, eGFR) and blood lipids indexes (TC, TG) were not significantly different from those before treatment (P > 0.05).

**TABLE 2:** Laboratory test results before and after treatment.

Project	Experimental group				Control group			
	Baseline	24 weeks	Statistic	p-value	Baseline	24 weeks	Statistic	p-value
HBV DNA (IU/mL)	1110 (300,325000)	0 (0,17)	*Z* = 4.860	0.000	852 (40.5,111000)	0 (0,12)	*Z* = 4.458	0.000
HBsAg(IU/mL)	877 (287,1794)	606 (168,1095)	*Z* = 3.754	0.000	470 (176,1853)	292 (82,432)	*Z* = 3.377	0.001
ALT (U/L)	32 (24,46)	23 (18,26)	*Z* = 3.786	0.000	23 (17,77)	19 (16,30)	*Z* = 2.071	0.038
AST (U/L)	48.1 ± 25.7	30.7 ± 11.4	*t* = 3.847	0.001	39 (27,65)	29 (25,33)	*Z* = 2.921	0.003
TBIL (μmol/L)	25.15 ± 14.19	18.45 ± 10.55	*t* = 2.822	0.008	21.9 (15.5,28.0)	19.9 (15.0,26.5)	*Z* = 1.258	0.209
Cr (μmol/L)	67.0 (54.0,82.0)	68.0 (56.7,81.0)	*Z* = 0.260	0.795	60.27 ± 13.16	58.88 ± 12.12	*t* = 0.633	0.533
eGFR (mL/min/1.72m^2^)	92.8 (83.2,96.1)	90.8 (83.2,95.5)	*Z* = 0.147	0.883	96.20 (92.60,99.25)	93.25 (90.70,98.20)	*Z* = 1.702	0.089
TC (mmol/L)	3.99 (2.93,4.59)	3.95 (3.29,4.59)	*t* = 0.903	0.375	5.08 (4.09,5.52)	4.77 (4.08,5.27)	*Z* = 1.607	0.108
TG (mmol/L)	0.95 (0.75,1.23)	1.07 (0.76,1.41)	*t* = 0.923	0.365	1.33 (1.12,1.59)	1.43 (0.95,1.82)	*Z* = 1.121	0.262

#### 3.2.2 Virologic response

HBV DNA was detected at baseline in all patients in the experimental group, with a median value of 1110 (300,325000) IU/mL. After 24 weeks of TMF treatment, the HBV DNA significantly decreased (P = 0.000), resulting in a VR rate of 70.97%. The 27 patients in the experimental group did not achieve VR after 24 weeks of treatment, those patients with an average age of 73.67 ± 4.67 years, which was marginally elevated compared to the mean of all individuals in the experimental cohort, and the level of serum viral load was 562000 (332,4745000) IU/mL. Of the 27 patients who did not achieve VR, 21 were treatment-naive patients and 6 were treatment-experienced patients. The VR rate was 63.15% in the initial treatment group and 83.33% in the treated group, and there was no substantial difference between the two groups (P = 0.215). (Tables 2).

The experimental group was subdivided into HBeAg-positive and HBeAg-negative cohorts, with a 24-week VR rate of 73.91% in the HBeAg-negative group, slightly exceeding the 62.50% observed in the HBeAg-positive group; however, the difference lacked statistical significance (P = 0.161).

#### 3.2.3 HBeAg and HBsAg

**FIGURE 2:**
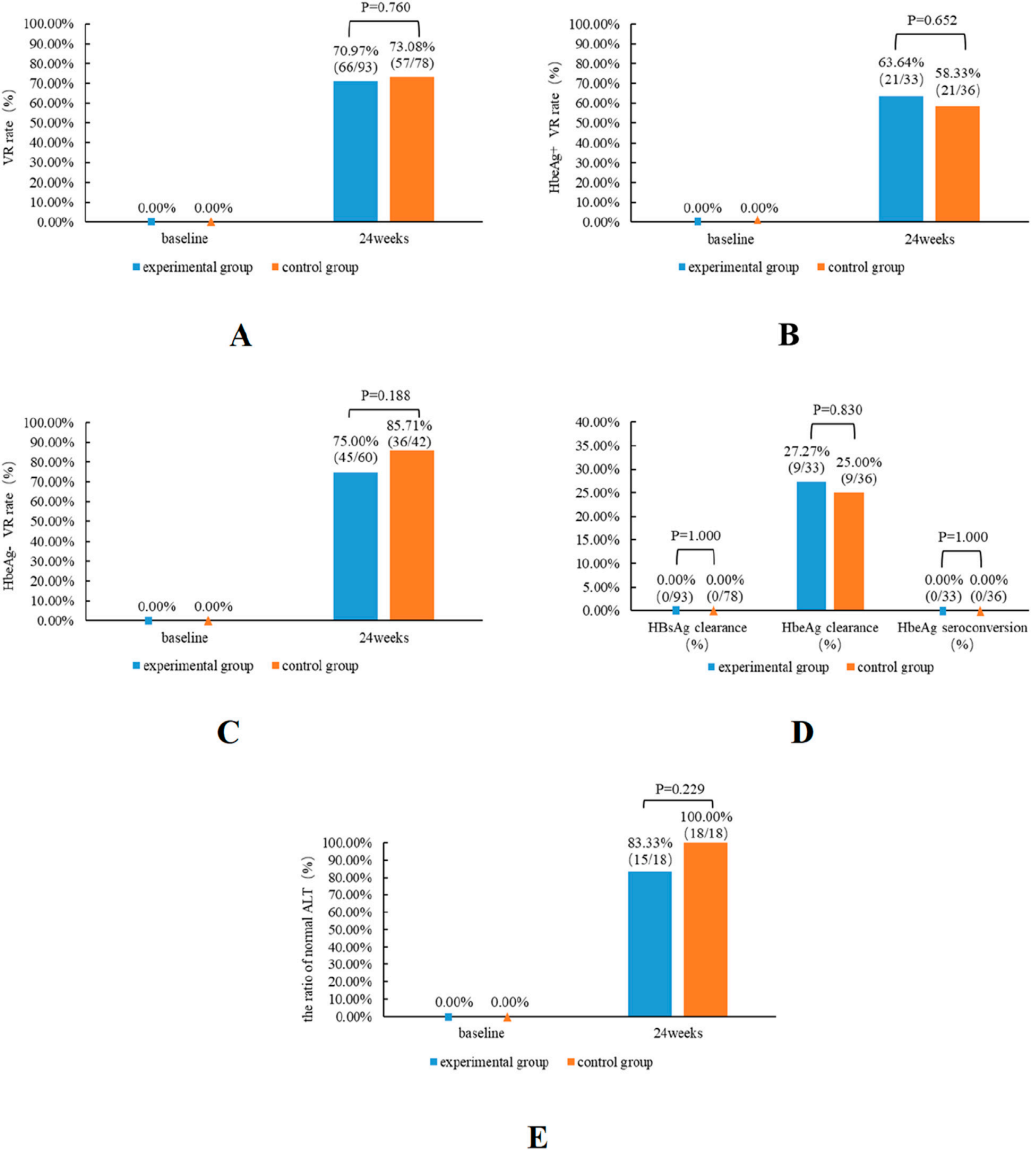
Antiviral effectiveness of TMF and TAF in patients from the experimental and control groups. The impact of virologic response rates in both patient groups **(A)**, HBeAg-positive patients **(B)** and HBeAg-negative patients **(C)**, subsequent to 24 weeks of therapy. The effects of HBsAg clearance, HBeAg clearance, and HBeAg seroconversion in both groups of patients **(D)** subsequent to 24 weeks of therapy. The impact of the normal ALT ratios in both patient groups **(E)**.

Following 24 weeks of TMF treatment, HBsAg levels in the experimental group decreased from 877 (287, 1794) IU/mL to 606 (168, 1095) IU/mL with statistical significance (P = 0.000). However, HBsAg seroconversion did not manifest in all patients within the experimental group. Within the experimental cohort, 33 HBeAg-positive patients, 9 patients had HBeAg clearance; the rate of clearance was 27.27%, but no patients had HBsAg seroconversion. (Tables 2; Figure 2).

#### 3.2.4 The ratio of ALT normalization

The baseline ALT level of the experimental group was 32 (24, 46) (U/L), and after 24 weeks of TMF treatment, the ALT level was 23 (18, 26) (U/L); the differentiation was a statistical difference (P = 0.000). The rate of ALT normalization was 83.33% in patients with abnormal baseline ALT after TMF treatment for 24 weeks. (Figure 2).

### 3.3 The safety profiles of TMF

#### 3.3.1 Renal function

Alterations in Cr and eGFR levels were compared with baseline data during the 24-week follow-up period. No substantial variation was observed in the Cr index pre- and post-treatment (P = 0.795). The eGFR was 92.8 (83.2, 96.1) mL/min/1.72 m^2^ at baseline and 90.8 (83.2, 95.5) mL/min/1.72 m^2^ after 24 weeks of TMF treatment, and the difference was no statistical difference (p = 0.883). The baseline eGFR of patients with abnormal baseline eGFR (<90 mL/min/1.72 m^2^) was 83.2 (67.7, 86.1) mL/min/1.72 m^2^, and 83.4 (64.3, 85.7) mL/min/1.72 m^2^ after 24 weeks of TMF treatment. No substantial difference was observed before and after treatment (P = 0.222) (Tables 2).

#### 3.3.2 Blood lipids

In our investigation, blood lipids mostly comprised total TC and TG. The TC level in the experimental group was 3.99 (2.93, 4.59) mmol/L at baseline and 3.95 (3.29, 4.59) mmol/L after 24 weeks of TMF treatment, and the disparity was statistically insignificant (P = 0.375). The TG level was 0.95 (0.75, 1.23) mmol/L at baseline and 1.07 (0.76, 1.41) mmol/L after 24 weeks of TMF treatment, and the difference was no statistical difference (p = 0.365) (Table 2).

### 3.4 Comparative efficacy and safety of TMF *versus* TAF

#### 3.4.1 Comparison of effectiveness

In terms of laboratory data, there was no notable difference at baseline. (P > 0.05), except the date of Cr, eGFR, TG, and TC. After 24 weeks of treatment, no substantial differences in HBV DNA, HBsAg, ALT, AST, and TBIL between the experimental group and the control group were observed (P > 0.05). Except for TBIL in the control group, the other laboratory indexes were markedly reduced compared to pre-treatment levels (P < 0.05) (Tables 2, 3).

**TABLE 3:** Laboratory test results for 24 weeks treatment.

Project	Experimental group	Control group	Statistic	p-value
HBV DNA (IU/mL)	0 (0,17)	0 (0,12)	*Z* = 0.384	0.701
HBsAg(IU/mL)	606 (168,1095)	292 (82,432)	*Z* = 1.795	0.073
ALT (U/L)	23 (18,26)	19 (16,30)	*Z* = 0.297	0.766
AST (U/L)	29 (24,35)	29 (25,33)	*Z* = 0.233	0.816
TBIL (μmol/L)	18.3 (8.2,26.1)	19.9 (15.0,26.5)	*Z* = 0.889	0.374
Cr (μmol/L)	68.0 (56.7,81.0)	58.0 (46.0,72.5)	*Z* = 2.478	0.013
eGFR (mL/min/1.72m^2^)	90.80 (83.20,95.50)	93.25 (90.70,98.20)	*Z* = 2.316	0.021
TC (mmol/L)	4.02 ± 1.12	4.58 ± 1.18	*t* = 1.686	0.099
TG (mmol/L)	1.07 (0.75,1.41)	1.42 (0.95,1.82)	*Z* = 2.153	0.031

The experimental group’s VR rate was slightly lower than the control group’s (70.97% vs. 73.08%); however, no significant difference was observed between the two groups (P = 0.760). Among the HBeAg-positive patients, the VR rate of the experimental group was slightly higher than that of the control group. And among the HBeAg-negative patients, the VR rate of the experimental group is marginally superior to that of the control group. However, regardless of HBeAg status, no substantial difference existed between the experimental group and the control group. Compared with the baseline data, the proportion of normal ALT was 80.65% in the experimental group and 76.92% in the control group. Following 24 weeks of treatment, the percentage of normal ALT levels was 96.77% in the experimental group and 100% in the control group. No significant difference was observed in the ALT normalization rate between the two groups at week 24 (P = 0.229). At baseline, the experimental group comprised 33 HBeAg-positive patients, while the control group included 36 HBeAg-positive patients, with 9 cases of HBeAg clearance in each group, and the HBeAg clearance rate was 27.27% in the experimental group and 25% in the control group (Figure 2).

#### 3.4.2 Comparison of safety

**FIGURE 3:**
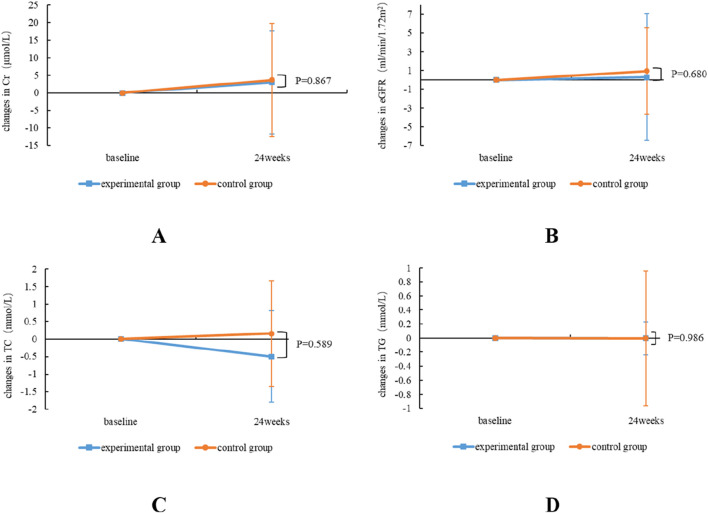
Comparative alterations in renal and blood lipids safety profiles between TMF and TAF in both experimental and control group subjects. Alterations in Cr **(A)** and eGFR **(B)** at week 24 post-treatment; TC **(C)**, and TG **(D)** at week 24 post-treatment. All bars are represented as $\bar{x}$ ±*s*.

During 24 weeks treatment, the renal function of both the experimental group and the control group exhibited a marginal increase in Cr, and eGFR showed an improvement trend in both groups; however, there was no notable difference in the alterations in renal function indices between the two groups (P > 0.05). In terms of blood lipids, TC and TG in the experimental group were slightly improved, but TC was slightly increased in the control group, and there was no notable difference between the two groups for the alterations in the aforementioned blood lipid indices (P > 0.05) (Table 3; Figure 3).

## 4 Discussion

In the retrospective real-world study, elderly patients diagnosed with decompensated hepatitis B cirrhosis received treatment with TMF for 24 weeks, and the findings indicated that TMF was particularly efficient in inhibiting HBV replication in this population. All patients had a substantial reduction in their HBV DNA levels, and we found that nearly 71% of elderly patients diagnosed with decompensated hepatitis B cirrhosis achieved a virologic response after treatment for 24 weeks. Additionally, the VR rate was marginally elevated in HBeAg-negative individuals compared to HBeAg-positive patients, which is consistent with the results observed by Liu et al. in a 96-week clinical study (Liu et al., 2023). Based on the structural features of TMF, the drug has smaller adverse drug reactions on kidney function. And adverse drug reactions on kidney function were not observed in elderly patients diagnosed with decompensated hepatitis B cirrhosis treated with TMF for 24 weeks. Patients with underlying kidney function abnormalities also did not experience further deterioration of kidney function after taking the drug. Furthermore, this study evaluated the efficacy and safety of TMF and TAF in elderly patients diagnosed with decompensated hepatitis B cirrhosis, and no statistical differences were observed.

A phase III clinical research study demonstrated that following 48 weeks of treatment, the VR rate of HBeAg-positive patients was 50.2%, whereas for HBeAg-negative patients it was 88.9%. After treatment for 96 weeks, the VR rate of HBeAg-positive patients was 70.80%, and that of HBeAg-negative patients was 93.90% (Liu et al., 2023). The findings at 24 weeks of this study indicated that the VR rate of elderly HBeAg-positive patients diagnosed with decompensated hepatitis B cirrhosis was 66.4%, which was higher than the 48-week TMF phase Ⅲ clinical data but lower than the 96-week TMF phase Ⅲ clinical data. The VR rate in elderly HBeAg-negative patients with decompensated hepatitis B cirrhosis was 75.00%, which was lower than the 48-week and 96-week TMF phase Ⅲ clinical data, also lower than the 48-week real-world study data (Rong et al., 2025). In the TDF clinical study, it was found that the virologic response rate gradually increased with the extension of treatment time (Lim et al., 2019; Lee et al., 2018). Both TMF and TDF are eventually metabolized into tenofovir (TFV) to play an antiviral role in the human body. Therefore, it is reasonable to observe that the virologic response rate at 24 weeks is lower than that at 48 and 96 weeks in the TMF study. In our study, the VR rate of HBeAg-positive patients at 24 weeks was higher than that of the 48-week TMF phase III clinical data, which was caused by the following 2 reasons: 1. The cohort of HBeAg-positive patients in our investigation was limited; 2. The initial HBV DNA levels of the participants were low, with 36.36% of those exhibiting baseline HBV DNA <100 IU/mL being HBeAg positive.

The 24-week VR rate of TMF and TAF, in the experimental group was 70.97%, which was marginally below 73.08% in the control group. This pertained to the marginally elevated percentage of patients with HBV DNA baseline <100 IU/mL in the control group, which was 19.35% in the experimental group and 23.08% in the control group. Nonetheless, there was no notable disparity in the VR rate between the two groups at 24 weeks. Furthermore, the assessment of antiviral efficacy according to HBeAg status indicated that the VR rate was diminished in the HBeAg-positive cohort in our investigation, which is also consistent with the data from the phase Ⅲ clinical trial. Regardless of HBeAg status, no significant difference in VR rate was seen between the experimental group and the control group. In our study, there was no increase in HBV DNA level compared with baseline after treatment for 24 weeks within the experimental group and the control group.

The goal of CHB treatment is to pursue clinical cure. However, HBsAg clearance is very difficult. A clinical study showed that the median rate of serum HBsAg reduction within 5 years in CHB patients treated with ETV was 0.125 log10 IU/mL/year (Seto et al., 2014). The findings of this investigation were analogous to those of prior research. After 24 weeks of treatment, HBsAg decreased slightly in both the experimental group and the control group, and no patient had HBsAg clearance in both groups. No significant difference was seen in the HBsAg clearance rate or HBsAg levels between the two groups at baseline and treatment for 24 weeks. Like other oral drugs that treat HBV infection, TMF is also difficult to achieve HBsAg clearance or seroconversion. For HBeAg-positive CHB patients, spontaneous or drug-induced HBeAg clearance has great clinical significance and is considered a milestone in the CHB treatment process. The TMF phase Ⅲ clinical trial showed that the HBeAg clearance rate at 48 weeks and 96 weeks was 17.2% and 27.0%, respectively. In our study, the HBeAg clearance rate at 24 weeks was 27.27%, which was slightly higher than in the clinical trial, attributed to the limited number of HBeAg positive patients included in our study, and 15 HBeAg positive patients were treated; the 15 patients were associated with lower HBeAg levels at baseline. There were both 9 patients with HBeAg clearance in the two groups, and 60% of the patients with HBeAg clearance were treated.

Previous studies have confirmed that TAF has an advantage in ALT normalization rate. Our study indicated that the ALT normalization rate was numerically lower in the experimental group compared to the control group; however, no significant difference was observed between the two groups. This is consistent with the study of Zhang (Zhang Q. et al., 2024). Because of the short observation time in this study, we cannot conclude which treatment is better for achieving ALT normalization.

In our study, after 24 weeks of TMF treatment, AST and TBIL were significantly lower than the baseline, and the differences were statistically different compared to the baseline. The findings indicated that TMF significantly improved liver function. In the control group, AST and TBIL levels were significantly lower than prior to treatment, with no notable difference observed between the two groups.

In recent years, the risk of renal injury and dyslipidemia caused by anti-HBV drugs of the TFV class has become the focus of clinical research (Milinkovic et al., 2019; Lin et al., 2024). Therefore, in terms of the safety of TMF, our study focuses on Cr, eGFR, TC, and TG of enrolled patients. The phase Ⅲ clinical trial of TMF at 96 weeks showed that eGFR and Cr decreased slightly. However, in our study, we observed a slight increase in Cr and eGFR compared with baseline, no significant difference was observed between pre-treatment and post-treatment measurements. In patients with abnormal eGFR, the mean eGFR had no change after 24 weeks of treatment compared with baseline. The above data indicate that TMF has well renal safety for patients. In comparison to the control group, the experimental group exhibited a marginally lower increase in eGFR; however, there was no statistical difference in renal function improvement between the two groups. Similar to the results of renal function, patients treated with TMF also had a slight improvement in blood lipids from baseline to week 24. Compared with baseline, TC decreased by 0.5 ± 1.30 mmol/L and TG decreased by 0.006 ± 0.23 mmol/L, but the difference was no statistical difference. In our study, we did not observe the effect of TMF on blood lipid in TMF phase Ⅲ clinical trials. The experimental group blood lipid levels were not different from the control data, too. The above safety laboratory data show that TMF has well kidney and blood lipid safety, which aligns with the data observed by other studies (Li et al., 2023; Zhang JS. et al., 2024; Chen et al., 2024; Peng et al., 2023).

## 5 Strengths and limitations

This is a retrospective cohort study observing the use of TMF *versus* TAF in elderly patients diagnosed with decompensated hepatitis B cirrhosis in the real world, which proves that TMF has well antiviral effect and safety, and there is no statistical difference between TMF and TAF. At present, the observation objects of the articles on TMF published are mainly young and middle-aged CHB patients. Compared with these studies, our study population is more special, which is elderly patients with decompensated hepatitis B cirrhosis, making up for the current research gap on this population. The methods employed are not innovative, and the study design resembles that of numerous existing articles; however, this study addresses the gap in TMF phase III clinical trials and enhances the understanding of TMF research. Additionally, there are several other limitations. This study is a single-center, retrospective analysis characterized by a limited sample size and a brief follow-up period. TMF is a new prodrug of tenofovir that was introduced in China in June 2021. Because of the limited duration on the market, relatively few patients were treated with TMF, and elderly patients with decompensated hepatitis B cirrhosis were more limited, which resulted in the limited sample size of this study. Secondly, because the data of serum calcium and serum phosphorus could not be obtained, the influence of TMF on the relevant results could not be analyzed. Thirdly, there is a lack of biomarkers for skeletal abnormalities, which is difficult to obtain because patients in the real world often have a follow-up period of more than 24 weeks. Long-term observational studies with large sample sizes and comprehensive indicators are essential for the reasons outlined.

## 6 Conclusion

This 24-week retrospective cohort study demonstrated that TMF exhibits superior anti-HBV efficacy in elderly patients diagnosed with decompensated hepatitis B cirrhosis while not significantly impacting renal function or blood lipids levels. TMF demonstrated non-inferiority to TAF in terms of both anti-HBV efficacy and safety. Nonetheless, the limited sample size and brief follow-up duration necessitate additional research involving a larger cohort and extended follow-up to validate our findings.

## Data Availability

The original contributions presented in the study are included in the article/supplementary material, further inquiries can be directed to the corresponding author.
